# Effect of Residue Acrylic Monomers in Synthesized Solvent-Free Photoreactive Pressure-Sensitive Adhesives on the Main Properties of Transfer Tapes Applied to Joining Wooden Elements

**DOI:** 10.3390/ma16247563

**Published:** 2023-12-08

**Authors:** Zbigniew Czech, Marcin Bartkowiak, Tomasz Krystofiak

**Affiliations:** 1 Department of Chemical Organic Technology and Polymeric Materials, Faculty of Chemical Technology and Engineering, West Pomeranian University of Technology in Szczecin, Piastów Ave. 42, 71-065 Szczecin, Poland; psa_czech@wp.pl (Z.C.); mbartkowiak@zut.edu.pl (M.B.); 2 Department of Wood Science and Thermal Techniques, Faculty of Forestry and Wood Technology, Poznań University of Life Sciences, Wojska Polskiego 28, 60-637 Poznań, Poland

**Keywords:** photopolymerization, photo-crosslinking, UV radiation, transfer tapes, photoreactive prepolymers, residue monomers, tack, peel adhesion, shear strength, shrinkage

## Abstract

This publication describes the influence of residue monomers in synthesized pressure-sensitive adhesives based on acrylics on their main properties—tack, peel adhesion, shear strength and shrinkage—in the form of transfer tapes used for joining wooden elements in the furniture industry. The discussed carrier-free adhesive tapes are synthesized via photo-crosslinking and photopolymerization with UV radiation of the photoreactive prepolymers sandwiched between two adhesive siliconized polyester films. The simultaneous crosslinking and polymerization processes carried out under UV lamps placed simultaneously above and below the crosslinked photoreactive polymer layer lead to the production of a carrier-free adhesive film. The preliminary target of these studies was to investigate how the intensity of UV radiation and the time of its exposure affect the viscosity of the photoreactive compositions and the content of unreacted monomers in them. Next, the influence of the crosslinking agent concentration and UV irradiation time on the content of unreacted monomers after the crosslinking process was tested. The last step of the studies was the investigation of the influence of the residue monomer concentration on the application properties of the obtained pressure-sensitive adhesive layers. The typical PSA application properties were tested on the wood samples: tack, peel adhesion, shear strength (cohesion) and shrinkage.

## 1. Introduction

Polymers characterized by a low Tg (glass transition temperature) below about −20 °C and an amorphous structure are predisposed for the manufacturing of PSA (pressure-sensitive adhesives) [1]. Since the introduction of pressure-sensitive acrylic adhesives over half a century ago, they have been successfully applied in many fields of industry. PSAs are used in self-adhesive tapes [2,3,4,5,6,7,8,9,10], labels [11,12,13,14,15,16,17], signs and markings, protective films [12,14,15], assembly operations [4,11,12,18,19,20,21,22], as well as in dermal dosage systems for pharmaceutical applications [2,6,7,10,11,12,17,23,24,25,26,27] and biomedical electrodes [28]. Over the last 60 years or so, the development of acrylic pressure-sensitive adhesives has made tremendous progress, so much so that both manufacturers of pressure-sensitive adhesive articles and their adhesive suppliers now use sophisticated equipment and precise methods to study pressure-sensitive adhesive performance: tack, adhesion and cohesion. [16,29,30,31]. In the case of protective films, very important is the shrinkage of pressure-sensitive adhesives after the crosslinking process. Three properties which are useful in characterizing the performance of pressure-sensitive adhesives are: tack (the initial adhesion), peel adhesion, measured at a 90° and 180° angle, and cohesion—shear strength (static or dynamic shear). The first parameter shows the ability of a PSA to adhere quickly without any additional pressure, the second is its ability to resist removal by peeling (important for removable labels, etc.) and the third is its internal consistency when shearing forces are exerted on the adhesive layer. The first two parameters are directly proportional to each other but are inversely proportional to the third. The performance of PSAs, such as tack, peeling and shear, are to a large extent determined by the method of polymerization, crosslinking process and last but not least the type and quantity of the crosslinking agents added to the acrylic PSA [4,7,12,17,27,32]. The common technologies for manufacturing pressure-sensitive adhesives include the following kinds of acrylic pressure-sensitive adhesives (PSAs): solvent-based acrylic PSAs—the adhesive compounds are polymerized in solvent and cast onto the web and the solvents are dried off after coating, leaving behind the functional adhesive [11,12,19,21,31,33]; water-borne acrylic dispersion PSAs—the adhesive compounds are polymerized in water and then applied to the web, and, next, the water is dried off, leaving behind the functional adhesive; solvent-free acrylic PSAs in the form of typical hot melts, LVS (low viscosity systems) and photoreactive prepolymers. Adhesives based on photoreactive prepolymers are coated at room temperature and crosslinked via the application of UV technology using UV lamps emitting UV-A and UV-C radiation [12,13,19,21,28,32,33,34,35]. The modern construction and furniture industry often uses double-sided self-adhesive mounting tapes. These are most often universal tapes for various surfaces. There are not many studies in the literature on self-adhesive materials dedicated to joining construction wood with different surfaces [36]. The use of structural self-adhesive joints in connecting wooden elements brings a few beneficial improvements, primarily quick application and clean joints. In addition, no solvents are used, which accelerates the achievement of the final strength of the joint and is beneficial for safe working conditions. We found it necessary to expand the current knowledge about the production of modern self-adhesive tapes for connecting wood.

## 2. Materials and Methods

### 2.1. Materials

The following acrylate monomers, as shown in Table 1, were used in the tests performed below.

### 2.2. Synthesis of Investigated Solvent-Free Photoreactive Acrylic Pressure-Sensitive Adhesives

The solvent-free polyacrylate-based photoreactive pressure-sensitive adhesives (PSAs) were synthesized in a 0.25-litre glass reactor with water cooling using UV radiation as a source of free radicals resulting from the photolytic degradation of the photoinitiator. The monomer mixture consisted of 35 wt.% 2-ethylhexyl acrylate (2-EHA), 25 wt.% 2-propylheptyl acrylate (2-PHA), 15 wt.% butyl acrylate (BA), 10 wt.% methyl acrylate (MA), 10 wt.% ethyl acrylate (EA), and 5 wt.% acrylic acid (AA). A 0.1 wt.% Omnirad 127, based on the weight of the monomer mixture, was used as the radical photoinitiator.

The photoreactive acrylic PSAs were synthesized using UV-initiated polymerization in a stirred reactor under an inert atmosphere (N_2_) according to the bulk radical polymerization method. A reactor of 0.25-litre capacity was equipped with a stirrer, nitrogen inlet, temperature sensor and vent. UV radiation from a lamp mounted near the reactor was used to initiate the reaction. A UV lamp was placed by the reactor wall at a distance of 50 mm. A lamp with a power of 11 W (Osram Dulux S BL UVA 11 W/78, Munich, Germany) equipped with a reflector provided the radiation intensity at a level of 2.5 mW/cm^2^ at a given distance. The irradiation time was changed in the range of 1 min to 5 min. After exposure to UV radiation, oxygen was added to the pre-polymerization product to stabilize it.

### 2.3. Viscosity of Synthesized Solvent-Free Photoreactive Acrylic PSAs

The viscosity of the synthesized acrylic PSAs was measured using a Rheomat RM180 from TA Instruments (New Castle, DE, USA) (formerly Rheometric Scientific Inc., Seattle, WA, USA), with spindle No. 3 at room temperature (23 °C). This is a type of rotational viscometer device that uses the Brookfield method, where the spindle is immersed into the liquid sample and rotated at a defined speed.

This method is commonly used in the PSA industry due to its speed and simplicity.

### 2.4. Free Monomers Concentration in Synthesized Acrylic PSA

The residual of monomers was determined using the gas chromatograph Varian CP-3800 (Palo Alto, CA, USA). A chromatographic column J&W DB-1 with a length of 30 m and an internal diameter of 0.25 mm was used. From the above PSAs, 0.1 μL of the gaseous phase samples were injected into the chromatographic column for analysis using a Hamilton 7001 syringe (Merck KGaA, Darmstadt, Germany).

### 2.5. The Coating Weight of Transfer Acrylic PSA and Type of Cover Material

The coating weight (basis weight) of a pressure-sensitive adhesive is defined as the thickness of the adhesive layer. This parameter influences essentially the performance of every PSA. During the study, the prepared adhesives were coated onto a 50 g/m^2^ siliconized polyester film, and after coating, covered with the same 50 g/m^2^ siliconized polyester film. The basis weight (coating weight) of the pure adhesive layer between the foils was maintained at 100 g/m^2^.

### 2.6. UV-Initiated Crosslinking

**Figure 1:**
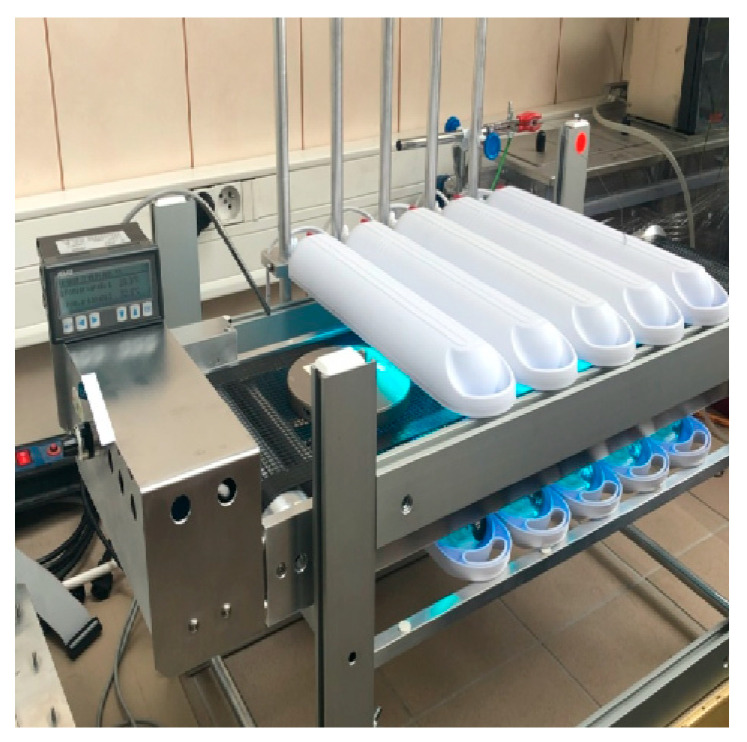
UV-initiated crosslinking of photoreactive transfer tape.

The coated layer of the photoreactive prepolymer is covered with a polyester adhesive film in order to exclude the so-called oxygen inhibition, negatively affecting the polymerization process. The “sandwich” obtained in this way is transferred from the coater to a device that allows the prepolymer layer to be illuminated on both sides using low-power UV-A lamps (Figure 1).

Photoreactive acrylic prepolymers are modified with the photoreactive crosslinking agent 1,6-hexanediol diacrylate (1,6-HDDA) in a concentration between 0.3 and 0.7 wt.%, are coated with a coat weight of 100 g/m^2^ directly onto the 50 µm thick siliconized polyester film, covered with the same 50 µm thick siliconized polyester film and, after that, crosslinked for 5 to 10 min under Philips low-Hg lamps with a UV dose of about 3 mW/cm^2^ (Figure 1). The UV irradiation is determined using an integrating radiometer Dynachem™ Model 500, available from Dynachem Corporation (Tustin, CA, USA).

### 2.7. Conditioning

Before the testing procedures, the adhesive-coated strips were kept at room temperature and 50% relative humidity for 7 days. During each measurement, three samples were used, and the given value of the tested property was the arithmetic mean of the achieved results from the three samples.

### 2.8. Measurement of Tack, Peel Adhesion, Shear Strength and Shrinkage

The influence of the concentration of the acrylate monomer residues, crosslinking agent (1,6-HDDA) amount and UV-crosslinking time on the PSA properties, such as tack, peel adhesion, shear strength and shrinkage, were determined following the international standards FINAT (FINAT—The Association For The European Self-Adhesive Labelling And Adjacent Narrow Web Converting Industries; finat.com (accessed on 15 June 2023)). The exact details can be found in FTM 9 (loop tack, measured at 23 °C), FTM 1 (peel adhesion at a 180° angle, measured at 23 °C) and FTM 18 (dynamic shear, measured at 23 °C and 70 °C).

The loop tack method measures the instantaneous adhesion of a loop of an adhesive-coated sample without external pressure in keeping contact (acc. FTM 9). According to another definition, the quick stick tack value is the force required to separate at a specific speed a loop of adhesive material brought into contact with a standard testing surface. A sample of PSA-coated material 1 inch (about 2.5 cm) wide and 7 inch (about 17.5 cm) long, in the form of a loop, is mounted into the jaws of the testing machine. A steel plate is mounted into the lower jaws. Then, the loop is lowered, causing brief contact between the adhesive and plate. The force reading in Newtons is recorded as the tape is peeled from the steel surface at a constant rate of 300 mm per minute. The loop tack test has the possibility of using various substrates, including wood substrates from Rocholl (Eschelbronn, Germany).

Peel adhesion at a 180° angle (acc. FTM 1) is the force required to remove a coated flexible pressure-sensitive adhesive sheet sample from a test panel measured at 180° on a wood surface as the rate of removal. More precisely, it is the force measured per width of the sample. A strip of PSA-coated material 25 mm wide and at least 175 mm long is bonded firmly to the surface of a clean steel test plate at least 12.7 cm. A 2 kg standard FINAT test roller is used to apply the strip to the plate. The free end of the coated strip is doubled back, nearly touching itself, so the angle of removal for the test is 180°. The free end is attached to the upper jaws of a tensile testing machine. The steel test plate is clamped into the lower jaws. The jaw separation rate is set at a constant rate of 300 mm per minute. Peel adhesion is measured after a specified contact time of 20 min, but for our studies, we set this time for only 2 min of contact between the PSA sample and substrate.

Shear strength is a measure of the cohesiveness (internal strength) of the pressure-sensitive adhesive according to the FTM 18 standard, at 23 °C and 70 °C. It is called also dynamic shear and describes the resistance of an adhesive sample joined to a test panel to shearing at a constant speed. It is measured as the maximum force required to remove the sample from a specified area in a direction parallel to the surface and at a constant rate of 5 mm per minute.

Shrinkage presents the percentage or millimeter change in the dimensions of the PVC foil covered with the PSA after PSA crosslinking. The PVC foil is attached to the glass and conditioned for 3 weeks at a temperature of 60 °C. Self-adhesive products with a shrinkage greater than 0.3% or 0.3 mm are not or only partially acceptable in the adhesive industry.

## 3. Results

### 3.1. Viscosity of Synthesized Solvent-Free Photoreactive Acrylic PSA

**Figure 2:**
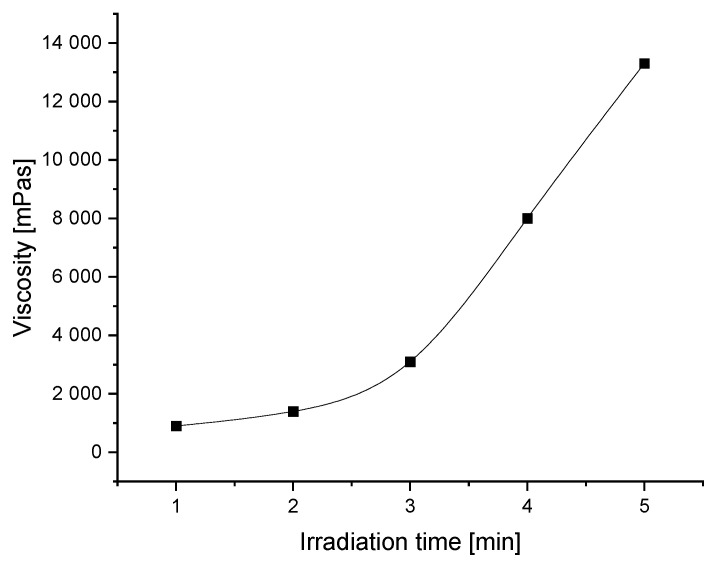
The viscosity of prepared photoreactive prepolymers as a function of irradiation time.

The exposure time is one of the parameters that allows for the adjustment of the viscosity of the prepolymer. Too low a viscosity leads to difficulties in coating since the prepolymer spills too quickly during the coating process. Too high a lightness, in turn, requires slower coating, which has a negative impact on the economics of production. In general, prepolymers with low viscosities of 1000 to 4000 mPa·s are used in the production of removable self-adhesive materials. Photoreactive prepolymers with viscosities of 8000 to 16,000 mPa·s are used in the production of classic self-adhesive materials, such as transfer adhesive tapes. As shown in Figure 2, the viscosity of the synthesized prepolymer increases with an increasing exposure time of the photoreactive mixture under the UV lamp.

During the first two minutes of irradiation, the viscosity increase is insignificant. After the next minute, faster change in this parameter can be observed. Particularly, between the third and fourth minute of reaction, viscosity increases almost three times (from 3100 to 8000 mPa·s). From the third minute of the reaction, an almost linear and fast increase in viscosity can be observed. This is a critical range within the reaction time to control the final viscosity of the prepolymer, due to the requirements of the coating process—as mentioned above.

### 3.2. Free Monomer Concentration in Synthesized Acrylic PSA

**Figure 3:**
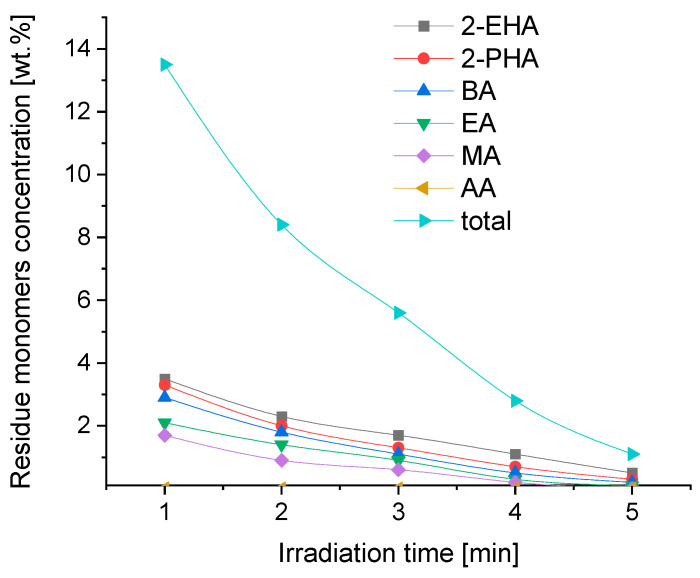
Concentration of residue monomers as a function of irradiation time.

The concentration of all unreacted monomers in the synthesized solvent-free prepolymer acrylic PSA was 13.5 wt.% and can be rated as relatively high (Figure 3). The exact concentrations of the used acrylate monomers corresponded with their reactivity, understood as the ability to radically polymerize with other monomers. And so, as can be seen in Figure 3, 2-ethylhexyl acrylate (2-EHA) and 2-propylheptyl acrylate (2-PHA) are characterized by the lowest reactivity. As the alkyl substituent decreases, the reactivity of the acryl alkylates increases. Thus, methyl acrylate is more reactive than ethyl acrylate, which in turn is more reactive than butyl acrylate. The most reactive monomer is acrylic acid, the concentration of which, as an unreacted monomer, is 0 wt.%. Acrylic acid with an extremely high reactivity was not found in the synthesized PSAs.

As can be observed in Figure 3 and also in Figure 2, an acceptable reaction time is above 4 min for the tested composition of monomers. After this reaction time, the viscosity is acceptable, as well as the residue monomer concentration. However, it is preferred to conduct the reaction for longer—up to 5 min—because a significant decrease in the concentration of residues can be observed (ca.2.5 times). Moreover, the viscosity is still acceptable for PSA coating.

### 3.3. UV-Initiated Crosslinking of Photoreactive Prepolymers

**Figure 4:**
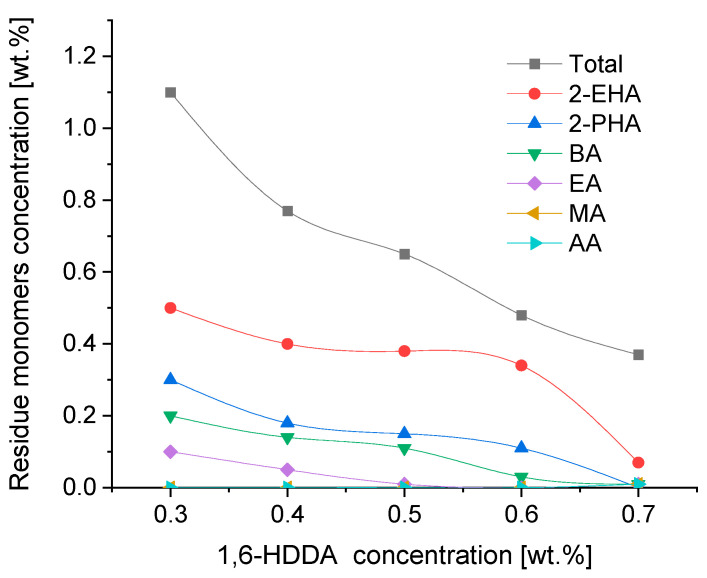
Residue monomer content as a function of 1,6-HDDA concentration.

Figure 4 shows the effect of the concentration of the difunctional monomer 1,6-hexanediol diacrylate used as a crosslinking agent in a concentration between 0.3 and 0.7 wt.% on the residue of the unreacted monomers in the photo-crosslinking process of acrylic adhesive films with a thickness of 100 g/m^2^ (ca. 100 µm), in this particular case, photo-crosslinking transfer adhesive films under a UV lamp for 5 min.

The use of photoreactive crosslinking compounds in the form of multifunctional acrylates, in this case, 1,6-hexanediol diacrylate (1,6-HDDA), is intended to obtain the appropriate internal strength (cohesion) of self-adhesive layers crosslinked under a UV lamp. During the increase in the cohesion of the UV-crosslinked adhesive films, there is also an increase in the conversion of the acrylate monomers used, and thus a decrease in the content of unreacted monomers. As shown in Figure 4, the increase in the concentration of the difunctional monomer 1,6-HDDA positively affects the reduction in the residue monomers used in the polymerization process of the photoreactive prepolymer. A further increase in the concentration of 1,6-HDDA, due to the increased degree of crosslinking of the adhesive film, leads to a decrease in its flexibility, which in turn leads to the deterioration of its self-adhesive properties, such as tack and peel adhesion.

### 3.4. UV-Initiated Crosslinking According to Crosslinking Time

The duration of the UV curing of the photoreactive prepolymer used for the production of transfer adhesive films is an important parameter influencing their final applicable properties. Too short a crosslinking time under the UV lamp causes the non-reaction of all, or almost all, of the monomers used for the production of transfer adhesive films. The remains of the unreacted monomers, apart from causing an unpleasant odor, may negatively affect the properties of the adhesives, such as tack, peel adhesion, shear strength and shrinkage.

**Figure 5:**
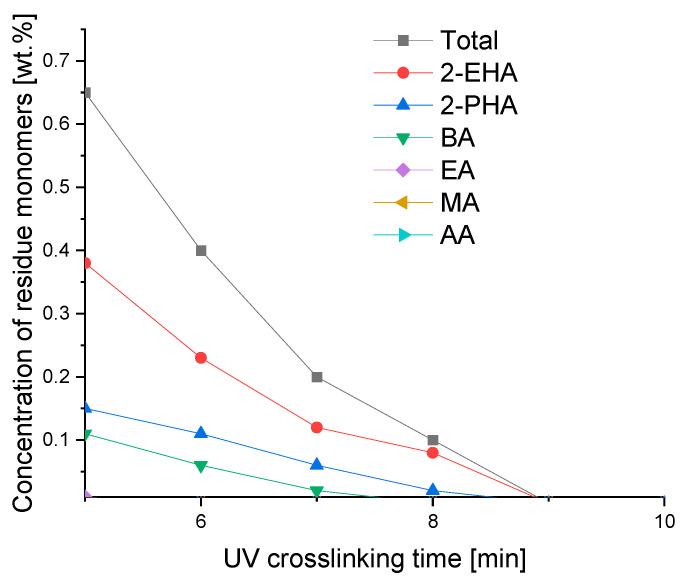
Residue monomers content as a function of UV crosslinking time.

As shown in Figure 5, the crosslinking time of the adhesive films is important for reducing the concentration of classic monomers and, as in the case of more reactive monomers, for their complete conversion. The more reactive ones include acrylic acid (AA) and methyl acrylate (MA), where after 5 min of UV crosslinking time, the adhesive layer does not contain them. After 6 min of exposure to the UV lamp, no ethyl acrylate (EA) is observed, and after 8 min, no butyl acrylate (BA). After 9 min, the unreacted 2-ethylhexyl acrylate (2-EHA) and 2-propylheptyl acrylate (2-PHA) disappear. A crosslinking time of 10 min under a UV lamp allows us to obtain, free of unreacted monomers, a high-quality self-adhesive transfer tape.

### 3.5. Effect of Residue Monomers on Tack, Peel Adhesion, Shear Strength and Shrinkage

**Figure 6:**
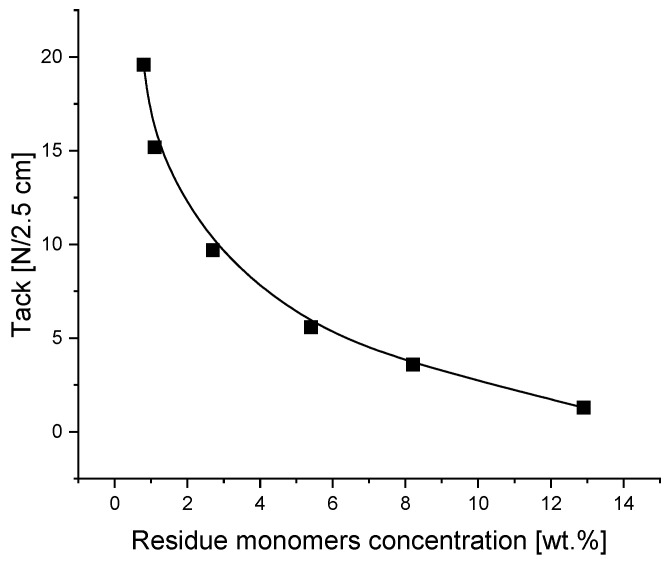
Tack of transfer tapes as a function of the concentration of residue monomers.

**Figure 7:**
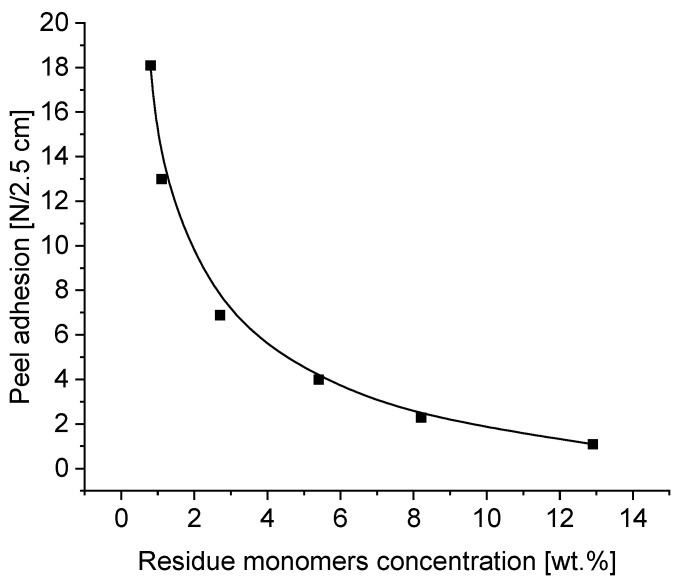
Peel adhesion of transfer tapes as a function of residue monomer concentration.

**Figure 8:**
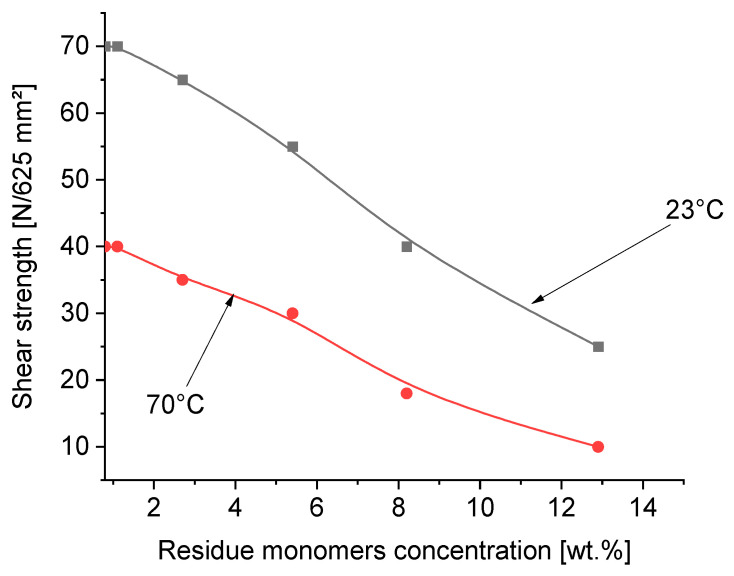
Shear strength of transfer tapes as a function of residue monomer concentration.

**Figure 9:**
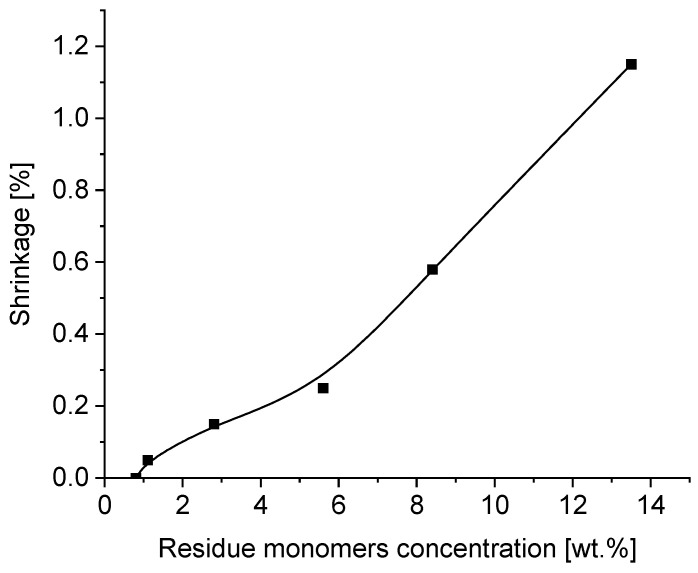
Shrinkage of transfer tapes as a function of residue monomer concentration.

The aim of this part of the publication was to investigate the influence of residue unreacted acrylic monomers in transfer self-adhesive tapes based on photoreactive acrylic PSAs on their important properties, such as tack, peel adhesion, shear strength and shrinkage. The details of the tests and the obtained results on tack, peel adhesion, cohesion and shrinkage on a wooden substrate obtained from the German company Rocholl are presented in Figure 6, Figure 7, Figure 8 and Figure 9: tack in Figure 6, peel adhesion in Figure 7, shear strength in Figure 8 and shrinkage in Figure 9, respectively.

Predictably, the presence of free unreacted acrylate monomers adversely affects the transfer tack of adhesive tapes to the wooden surface. Of course, this unfavorable tendency depends on the concentration of the residue monomers and is intensified by an increase in the concentration of unreacted monomers. The more unreacted monomers there are, the smaller the tack, also called initial adhesion. The increased content of unreacted monomers migrates faster than the smaller content of unreacted monomers to the surface of the wood, which logically justifies the reduction in tack. As shown in Figure 6, the highest tack value of 20 N was achieved with transfer adhesive tapes containing approximately 1 wt.% of residue monomers. A content of residue monomers above 2–3 wt.% has a very negative effect on tack and is unacceptable in the technology of joining wooden elements.

As in the case of tack, the presence of residue monomers in transfer adhesive tapes adversely affects adhesion, which is referred to as peel adhesion (Figure 7). As in the case of tack, the presence of residue monomers in transfer adhesive tapes adversely affects the adhesion, which is referred to as peel adhesion. Adhesion, measured as peel adhesion, depends on the contact time of the adhesive with the wooden substrate. In application conditions, the contact time of the transfer adhesive tape with the substrate to be glued very often does not exceed a few minutes. For this reason, the peel adhesion test was performed after 2 min.

As in tack testing, the measured values of peel adhesion decrease due to the presence of unreacted free residues. Although the measured values of adhesion are generally higher than the tack values, we also observe the negative impact of residue monomers on these values here. The remains of the unreacted acrylate monomers migrate from the inside of the adhesive film, thus worsening the adhesion of the transfer adhesive tape to the wooden substrate. As in the case of tack, peel adhesion on a wood substrate requires the use of adhesive tapes with less than 2 wt.% of residue monomers (Figure 7).

Standard shear strength resistance testing is carried out with a specified area of adhesive tape applied to a standard test surface, in this case, a wooden surface. The shear failure is the inability of the PSA layer to resist opposing stress. Any kind of task that is a measure of stress relaxation within the adhesives gives important data. A highly shear-resistant adhesive layer will resist tension, while an adhesive with poor shear resistance will shear quite rapidly. Figure 8 presents the shear strength of acrylic self-adhesive layers dependent on the free monomer concentration in the polymer layers.

The content of free unreacted acrylate monomers in self-adhesive tapes reduces the internal strength of the adhesive joint (cohesion), measured both at 23 °C and 70 °C. The cohesion values measured at 23 °C are obviously higher than at 70 °C. At 70 °C, the migration of residue monomers from the adhesive layer of the adhesive tape to the surface of the joined wooden elements is higher than at room temperature; therefore, the cohesion at 70 °C is lower than at 23 °C. Here, as in the case of tack and peel adhesion, the content of residue monomers in the range of 2–3 wt.% ensures an even higher strength of the bonded adhesive joint (Figure 8).

The shrinkage of transfer adhesive tapes is an extremely important parameter when bonding substrates with different coefficients of thermal expansion and different surface energies. It depends on many factors, in this particular case, on the amount of double bonds in the non-crosslinked and then in the crosslinked polymer in the form of an adhesive tape. Shrinkage is also affected by the unreacted monomer residues in the polymer after UV crosslinking.

Acrylic polymers have been successfully used as pressure-sensitive adhesives in various industries, and a property common to all acrylic PSAs, which negatively impacts their performance, is the shrinkage onto different surfaces (wood, steel, glass, etc.) after crosslinking. The shrinkage characteristics of solvent-free acrylic PSAs depending on the content of free monomers are shown in Figure 9. The best results for shrinkage under 0.05–0.2% were achieved for PSA layers containing no more than about 2–3 wt.% of free unreacted monomers. A shrinkage for PSAs greater than 0.3% is completely unacceptable.

## 4. Conclusions

Summing up, from the performed evaluation of the experiments referring to tack, peel adhesion, shear strength and shrinkage of the photoreactive solvent-free acrylic pressure-sensitive adhesives, by testing the self-adhesive acrylic layers containing various concentrations of residue unreacted free acrylate monomers discussed in this article, it can be concluded that the best results regarding the investigated properties were obtained for carrier-free films characterized by a coating weight of 100 g/m^2^ (100 µ), containing no more than 2–3 wt.% of residue monomers. When carrying out the synthesis of solvent-free photoreactive self-adhesive adhesives based on acrylates, it is not possible to obtain transfer (carrier-free) self-adhesive tapes free of unreacted monomers in the final product. The amount of unreacted monomer residues in the amount of about 13.5 wt.% corresponds to their reactivity, which is also dependent on the other monomers present in the mixture. In general, the reactivity of acrylate monomers decreases with an increase in their alkyl part, and thus increases with a decrease in their alkyl part. This is confirmed via GC analyses of mixtures of unreacted monomers, where 2-ethylhexyl acrylate, 2-propylheptyl acrylate and butyl acrylate predominate. As for the other acrylates used in research, such as ethyl acrylate and methyl acrylate, their concentration is relatively low. The lack of acrylic acid in the mixture of unreacted monomers indicates its high reactivity and thus complete conversion. The relatively high concentration of unreacted monomers in the self-adhesive photoreactive prepolymer results from the method of conducting bulk polymerization without the participation of a solvent. A few minutes are enough to obtain a photoreactive prepolymer with a specific viscosity that allows it to be coated. During this time, a prepolymer with a viscosity of about 2000 to 14,000 mPa·s is formed, which, due to the very short polymerization time, contains a relatively large concentration of unreacted monomers, often exceeding 10% in weight. Classic acrylic polymer pressure-sensitive adhesives synthesized in a solvent within 2–6 h contain up to 2–4 wt.% of unreacted monomers. The content of residue acrylic monomers can be reduced by increasing the time of irradiation of the monomer mixture using UV radiation, but only up to a certain point, when the increase in the viscosity of the obtained prepolymers makes it possible to coat it and obtain self-adhesive tapes. Residual monomers are also reduced during the crosslinking of the prepolymer into the polymer using a multifunctional acrylate, in this particular case, difunctional 1,6-hexanediol diacrylate (1,6-HDDA). Increasing the concentration of the photoreactive crosslinking compound increases the conversion of the polymers used in the polymerization, and thus reduces the content of unreacted acrylate monomers. An additional possibility in reducing the concentration of residue monomers is an increase in the crosslinking time under the UV lamp. After 6 min of crosslinking of the photoreactive prepolymer containing the photoreactive crosslinker, transfer adhesive tapes containing unreacted acrylate monomers with a maximum concentration of about 0.4 wt.% are obtained. Further crosslinking within 7–8 min allows for the reduction of the residue monomers to 0.1–0.2 wt.%. And after 10 min, transfer adhesive tapes are obtained with a concentration of unreacted monomers below 0.1 wt.%. When examining the influence of residue monomers within 1–3 wt.% on tack (Figure 6), peel adhesion (Figure 7), shear strength at 23 °C and 70 °C (Figure 8) and on shrinkage (Figure 9), it can be noticed that the limitation of residue monomers to about 1 wt.% does not have any significant effect on the application properties of transfer adhesive tapes used in the furniture industry. From the experimental results in the application of prepared carrier-free PSA tapes, the conclusion can be inferred that the use of acrylic PSAs with more than 2–3 wt.% of free monomers negatively influences all the evaluated properties of solvent-based acrylic PSAs, especially the cohesion tested at 23 °C and 70 °C. Synthesis of acrylic PSAs containing less than 1 wt.% of free monomers allows for an excellent tack, peel adhesion, shear strength and shrinkage performance to be reached.

## Figures and Tables

**Table 1:** Raw materials used for the synthesis of studied solvent-free acrylic PSAs.

Kind of Monomers	Abbreviation	Chemical Formula	Supplier
2-ethylhexyl acrylate	2-EHA	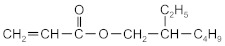	BASF (Ludwigshafen, Germany)
2-propylheptyl acrylate	2-PHA	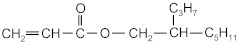	BASF (Ludwigshafen, Germany)
butyl acrylate	BA	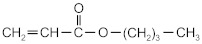	BASF (Ludwigshafen, Germany)
ethyl acrylate	EA	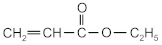	BASF (Ludwigshafen, Germany)
methyl acrylate	MA	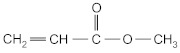	BASF (Ludwigshafen, Germany)
acrylic acid	AA	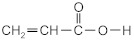	BASF (Ludwigshafen, Germany)

The radical photoinitiator used during the studies was Omnirad 127 from IGM Resins B.V. (Waalwijk, The Netherlands), CAS: 474510-57-1, with the chemical name 2-hydroxy-1-[4-[4-(2-hydroxy-2-methylpropionyl)benzyl)phenyl)-2-methylpropan-1-one. The photoreactive crosslinking agent used for PSA crosslinking was 1,6-hexanediol diacrylate (1,6-HDDA) (Merck KGaA, Darmstadt, Germany). CAS: 13048-33-4.

## Data Availability

The data presented in this study are available on request from the corresponding author. The data are not publicly available because currently they form part of an ongoing studies.
